# Teaching of cursive writing in the first year of primary school: Effect on reading and writing skills

**DOI:** 10.1371/journal.pone.0209978

**Published:** 2019-02-07

**Authors:** Cristina Semeraro, Gabrielle Coppola, Rosalinda Cassibba, Daniela Lucangeli

**Affiliations:** 1 Department of Education, Psychology, Communication, University of Bari “Aldo Moro”, Bari, Italy; 2 Department of Developmental Psychology and Socialization, University of Padova, Padova, Italy; Hangzhou Normal University, CHINA

## Abstract

There is increasing evidence that mastering handwriting skills play an important role on academic achievement. This is a slow process that begins in kindergarten: at this age, writing is very similar to drawing (i.e. scribbles); from there, it takes several years before children are able to write competently. Many studies support the idea that motor training plays a crucial role to increase mental representations of the letters, but relatively little is known about the specific relation between handwriting skills and teaching practices. This study investigated the efficacy of cursive writing teaching. The sample comprised 141 students attending eight classes of the first grade of primary school, all with typical development, not exhibiting any cognitive or sensory disabilities, nor displaying motor disorders that could significantly hinder the execution of the writing task. We tested whether the development of academic writing skills could be effectively supported by training strategies focusing on cursive writing. All rules and characteristics of the letters were explained by demonstrating the correct writing movements, based on the idea that movement learning becomes more valuable when children begin to connect the letters in order to write individual words. Growth models on pre-, post- and follow-up measures showed that performance on prerequisites and writing and reading skills were better overall among the children in the intervention group as compared to control group.

## Introduction

The research in the area of handwriting ability highlights an increase in graphical and visual-spatial difficulties in handwriting [1]. “Dysfluent writing” and “shape abnormality” are key characteristics of handwriting disorders described in the 5th edition of the Diagnostic and Statistical Manual of Mental Disorders of the American Psychiatric Association (DSM-5). The term “dysfluent writing” refers to less fluent (i.e., slower) handwriting, while the term “shape abnormality” refers to distortions of pressure and irregularities in the forms of letters. According to the official international diagnostic systems [2,3,4], visual-motor and visual-spatial difficulties in writing are manifestations of a motor development disorder (dysgraphy). Poor graph-motor skills may increase the risk of difficulties in the visual-motor and spatial components of writing; therefore, interventions aimed at supporting and enhancing graphic activity, a function not adequately recognized thus far [5], could represent a valid action to prevent graphical and spatial difficulties in writing, especially in the context of formal education.

At the time of entry into primary school, children use much of their cognitive energy to control the production of letters and the graphical aspects of writing. A significant portion of their time and cognitive energy is, in fact, invested in controlling down processes (for example, correct writing of letters), while few attentive resources remain available for more complex tasks, such as generating ideas, lexical access, management of cognitive activities, and orthographic review of the text [6].

The well-known “cognitive constraint” related to writing [7,8,9] suggests the importance of automating the production of letters and words during writing, so that children can direct their attentive resources to the more complex aspects of text production, such as the decoding of reading and the orthographic accuracy of writing [10,11]. Available findings support the idea that, in the second [11] and third grade of primary school [12], a considerable amount of variance in the quality of a written text can be attributed to the automation of the production of letters. Since the movements necessary for the production of letters are under voluntary control, if not automated they can represent a high cognitive cost for children in terms of attention, which in turn prevents them from performing higher order academic tasks such as composing or paying attention to spelling and grammar [13].

With this in mind, research has examined the efficacy of early interventions targeting handwriting or of spelling instruction for struggling writers in first grade and findings show increased output, improved sentence writing skills, and better writing quality for children benefitting of such support [14,15,11]. Similar gains in writing output and sentence writing skills were obtained when struggling writers in second grade were provided with extra spelling instruction [16,17]. The findings from the two studies reviewed above, altogether, indicate that handwriting intervention early in primary grades may be a critical factor in preventing writing difficulties, at least for children who do not master handwriting easily [18,19,20]. Graham and Harris [21] reviewed the evidence on the role of handwriting in children's development as writers. Consistent with the view that handwriting instruction is an essential ingredient in writing development, they found that handwriting skills, particularly handwriting fluency (i.e., the amount of text that can be written down correctly per minute), improve with age and schooling [22,23], and that individual differences in handwriting skills (most notably handwriting fluency) predict how much and how well children will write [24,25]. Thus, these findings underscore the importance of motor programs in supporting the development of writing skills in primary school children [26,27]. However, it is still unclear what should the training target in order to effectively promote writing development [28].

To promote better writing skills the choice of writing style seems to be fundamental [29]. Cursive style, besides being predictive of better writing skills, seems easier to learn for young children in primary school [29–31]. Let us consider the graphical features differentiating printed writing from cursive [32], as cursive and print are the basic types of handwriting that children learn in primary school [33,34]. The movements used for these types of handwriting can be generally classified as either discontinuous patterns (i.e., temporally consistent start-and-stop movements, as in printed handwriting) and continuous patterns (i.e., an emergent property of trajectory-throughout movements as in cursive handwriting). Consistency of movement in time and space has been claimed to be an important feature of “good” handwriting [35], and this is a distinctive characteristic of cursive writing.

It has been reported that younger-aged children have higher irregularity and inconsistency of movement and time when performing discontinuous loops than with continuous ones [36]; moreover, it seems that young children have more difficulty performing discontinuous handwriting patterns compared with continuous patterns [37].

We believe such differences require further attention. In printed characters, graphic movement is not continuous: the gesture stops, there are repeated stops and starts of the pencil and the motor process is broken. Instead, on the graph-motor plane, cursive is the writing style closest to the child’s natural movements. For example, if we think of scribbles, the first graphic charts of the child are curved and rotating and, by the age of 3, they tend to close the open shapes. Therefore, there is a spontaneous curvilinear and/or circular graphical tendency in writing processes; moreover, the first letter reproduced by children is usually the letter "O", which does not require a template. Lastly, while the reproduction of printed letters involves copying a static model composed of segments that must be plotted in a precise graphic direction, the letters in cursive connect to each other dynamically [1]. Spencer et al. [38] proposed different control mechanisms for continuous versus discontinuous movements. Performing discontinuous movements requires an explicit representation of the temporal goal (i.e., when to start and stop), whereas performing continuous movements does not require an explicit event-related timing process. The authors claimed that the explicit processes used in controlling temporal consistency of discontinuous movements involve the cerebellum. In contrast, implicit timing processes for continuous movements may not closely relate to the cerebellum. Behaviorally, temporal deficits in patients with cerebellum damage were restricted to discontinuous circle drawing [39]. It is known that the cerebellum develops more slowly over a longer duration (i.e., until about 16 years of age) than most of the subcortical and cortical areas [40] and it is especially vulnerable to developmental disorders [41]. Thus, there is also a neuropsychological rationale for believing that temporal control for discontinuous handwriting may be more challenging than for continuous patterns in young children due to the relatively slower cerebellum development. If this is the case, these findings suggest that teaching cursive writing should occur much earlier than it is typically done in the current education systems of most countries (e.g., Canada, France, the Netherlands). In particular, a study examined temporal consistency in continuous and discontinuous circle and line drawing in children from five to twelve years of age [31] and, in line with Spencer et al. [38], the explicit timing demands were lower in continuous drawing than in the discontinuous task. This study showed that young children had high temporal and spatial variability in discontinuous circle drawing but not in continuous circling, continuous line drawing, and discontinuous line drawing. Overall, these findings [20,25,29,35] suggest that the temporal control of discontinuous movements (i.e., printed handwriting) may be more challenging than that of continuous movements (i.e., cursive handwriting) and that these discontinuous movements are under voluntary control. This is a cost for children in terms of attention and cognitive resources. Therefore, cursive handwriting might be easier for young children to learn.

Despite this state of the art of the current literature, in Italy there is widespread belief among teachers that printed writing is easier for young children to learn than cursive writing. The guidelines of the Italian National Ministry of Education (MIUR) regarding teaching in primary school give no clear guideline about the timing and methods for teaching writing skills and teachers are free to make personal decisions in this regard (MIUR, reference legislation 2012 - Prot. n°5559 - 2012). Nevertheless, pedagogical models [25–29] have influenced the selection of global methods (use of printed characters for reading and writing) for read-write abilities. The predilection for printed characters is certainly not justified in light of what we know about the development of the child’s graph-motor skills, but exclusively on a perceptive basis.

Despite this, it is common practice to start teaching the printed style of writing and to move to cursive writing by the middle or end of the first year of primary school, continuing to give more attention to printed writing, especially for children with learning difficulties.

On this topic, the results of a study conducted by Morin and colleagues [35] are of particular interest: these scholars explored the existing relationship between three different methods of teaching writing (print writing only, cursive writing only, and print writing in the first grade of primary school with cursive in the second grade) and writing skills development (writing speed, spelling and text production) in a sample of children attending the second grade of primary school in Canada. Findings show that children who have learned to write using only the cursive style show superior performance in both spelling and syntax when compared to the other two groups. The teaching of both styles (first print, and then cursive in the second grade of primary school) does not favor the acquisition of automatic movements, resulting, therefore, in disadvantages compared to the cursive-only method.

All previously published studies have evaluated writing skills exclusively in terms of cognitive measurement, perceptive and graph-motor. Over the years, a broad range of studies have been developed pertaining, in particular, to the learning of writing skills for pre-school and primary school students without, however, deepening the issue of the methods used for teaching writing. To our knowledge, no previously published study has focused on using cursive as the primary type of writing in order to improve writing, reading and spelling skills in children at the start of primary school.

In order to fill this gap, we first aimed to evaluate the efficacy of a teaching program focuses on the effects of intensive cursive learning on the prerequisites of writing and reading. Secondly, we aimed to test the efficacy of the teaching program for the acquisition of writing and reading skills. More specifically, we propose a specific graph-motor training program and intend to evaluate the effect of the training on reading and writing skills at a distance of 6 months, comparing the children who benefited from the training with children who followed the standard programs used in Italian schools.

## Material and methods

### Participants

The sample comprised 141 students attending eight classes in the first grade (68 female—48.2%; 73 male—51.8%); age: *M* = 6.2, SD = .29) at four schools in the centre and suburbs of a city in the south of Italy. Selection criteria included Italian native speaker children, unidentified for cognitive or sensory disabilities and not displaying motor disorders which could significantly hinder the execution of the writing task. The eight classes were comparable in terms of: gender ratio (*χ*^2^ = 2.61, n.s.); age *t*(139) = 1.68, n.s.; measurement of socioeconomic status (parents’ years of education): mother’s educational level, *t*(127) = -.976, n.s. and father’s educational level, *t*(127) = -1.223, n.s.; teacher experience (all teachers were female with more than 15 years of teaching experience); and pre-test prerequisites of reading and writing skills (Table 1 and Table 2). The total sample was divided randomly into two sub-samples. An intervention group (TG) was made up of children from four classes (N = 73, F = 40 (54.8%), age: *M* = 6.16, SD = .28) who took part in the cursive training. The remaining four classes (N = 68; F = 28 (41.2%), age: *M* = 6.24, SD = .30) made up the control group (CG) and followed the standard programs of writing skills training (uppercase, lowercase, printed, and cursive writing were presented simultaneously). The study had the prior approval of the Local Ethics Committee of Department of Education, Psychology, Communication, University of Bari “Aldo Moro” (Committee: Andrea Bosco, Associate Professor in Psychometrics and Statistics, Antonietta Curci, Associate Professor in General Psychology, Valerio Meattini Full Professor in Theoretical Philosophy). For all children, parents signed an informed consent prior to the taking part to the protocol.

**Table 1:** Descriptive statistics and comparisons across the two sub-groups (control vs. intervention).

Variables	Control sub-sample	Intervention sub-sample	
	N = 68 (48.59%)	N = 73 (51.40%)	*Χ*^2^(1)
Males	40 (58.82%)	33 (45.20%)	2.61(n.s.)
	M (SD)	M (SD)	*t*(127)
Maternal Years of education	12.03 (3.12)	11.50 (3.06)	-.97 (n.s.)
Paternal Years of education	11.64 (3.23)	10.93 (3.36)	. -1.22 (n.s.)

**Table 2:** Means and standard deviations of prerequisites for reading and writing skills before training across the two sub-groups (control vs. intervention).

Task	Control sub-sample M(SD)	Intervention sub-sample M(SD)	*t*(*df*)
Semicircles	3.38 (3.32)	4.29 (2.49)	-1.85(140)
Recognition of Letters	1.09 (2.08)	1.03 (1.69)	-.18 (140)
Search of two letters	8.84 (6.20)	9.22 (6.33)	.36 (140)
Search of letters written in different ways	6.16 (2.85)	5.62 (2.53)	-1.20 (139)
Search of sequence of letters	18.54 (6.73)	17.15 (7.98)	-1.11 (140)
Handwriting Speed	42.17 (4.79)	43.44 (5.21)	1.50 (139)

*Note*. The statistic test was not significance.

#### Design overview

At pre-training (September, beginning of the school year: T0) two tasks to evaluate the prerequisites of reading and writing skills were administered. Subsequently, classes were randomly assigned to one of the two conditions: the intervention group, which took part in teaching sessions focused on cursive writing; and the control group, with classes following the traditional method of teaching writing skills. The training phase took place during the whole school year of first grade (September–May). The same tasks were administered post-training (in May, at the end of the school year: T1). In addition, in post-training, we administered a standardized battery of tests to evaluate reading and writing skills. Another six months later, prerequisites and reading and writing skills tests were administered a second time (T2) during a follow-up session (November).

#### Prerequisites assessment

Two tests were selected to assess the prerequisites:

1. Prerequisite of reading and writing skills PRCR-2/2009 (Batteria per la valutazione dei prerequisiti di letto-scrittura) [42]. Selective visual and left-right serial analysis tests were selected:

Semicircles: This task assesses the ability to analyze and remember graphic signs and their sequence; it also evaluates a visual memory of differently oriented signs.

Recognition of letters: This task examines visual analysis abilities.

Two-letter search: This task allows the evaluation of both discrimination and visual search abilities and the capacity to proceed from left to right, as well as the ability to make the short-term memory operational, all of which is fundamental to reading and writing learning.

Search of letters written in different ways: This task evaluates the ability to recognize a letter written in different allographs (uppercase, lowercase, printed and cursive) evaluating grapheme-phoneme conversion capacity.

Search of a sequence of letters: This task examines a child’s ability to look for a visual configuration sequentially, thus evaluating visual search abilities.

2. Test for the Evaluation of Writing and Orthographic Ability BVSCO-2 (Batteria per la Valutazione della Scrittura e della Competenza Ortografica-2) [43]. This task examines handwriting fluency when writing the sequence of letters “*LE*” for one minute (handwritten lowercase cursive characters *LE* praxis). The test involves the calculation of a measure of fluency: how many graphemes are written correctly in one minute.

All participants were evaluated through a collective administration.

#### Reading and writing skills assessment

Reading and writing skills assessment was conducted using the following tests:

1. MT battery for primary school [44], for the assessment of 3 parameters: fluency, accuracy and reading comprehension. Fluency and comprehension suggest two measures of reading ability, while accuracy of reading is represented by the number of errors committed.
2. Test for the Evaluation of Writing and Orthographic Ability BVSCO-2 (Batteria per la Valutazione della Scrittura e della Competenza Ortografica-2) [43]. This included two other tasks to evaluate handwriting fluency: writing the sequence of letters *UNO* (*ONE*) for one minute (*UNO* praxis) and writing the sequence of numbers *UNO-DUE-*, and so on (*ONE–TWO–…*) for one minute (Number praxis). The test involves the calculation of a measure of fluency: how many graphemes are written correctly in one minute. In addition, we selected another task concerning this battery to evaluate accuracy in writing during text dictation. This test measures spelling accuracy, represented by the number of errors committed.
3. Diagnosis of spelling disorders in developmental age, spelling to dictation test [45]: This consisted of two sections: dictation of (1) words and (2) pseudo-words. This test measures spelling accuracy as represented by the number of errors committed.

#### Training phase

The training lasted nine months, from September to May; forty sessions were managed by teachers who had been previously trained. Supervision was provided by psychologists who were expert in learning psychology (including the first author).

In applying this training to writing instruction, the first phase clarifies the conventions and characteristics of the letters, illustrating the necessary movements for their formation and verifying that each child has learned them (pre-graphism).

In the second phase, the child practices the production of letters, learning to control movements and trajectories. The aim of this phase is to produce graphemes carefully, respecting the proportions of letters, spaces, and lines of writing.

Each training session lasted about 90 minutes, with two weekly meetings. The experimental group practiced the cursive characters exclusively, while the control group practiced the two different types of writing (i.e. printed and cursive) simultaneously. These activities were carried out during teaching hours.

The sessions took place collectively, but the children worked on their own. At the beginning of the session, after a short period welcoming the children and making them feel comfortable (10 min.), and after leading review activities of the materials and activities presented in the previous session (10 min.), the teacher presented the new activities on the blackboard (20 min.). Each child practiced on their own cards for the various activities proposed (30 min.). After each card had been completed and coloured, children put the materials in a personal folder (10 min.). The last part of the lesson involved a blackboard exercise focusing on the materials presented during the session (10 min.). In order to consolidate learning, after every ten sessions there was a review lesson of the work done in the previous sessions. Children spent about 70 minutes per week writing in cursive, in accordance with the authors who have stated that handwriting should be taught systematically in short sessions several times a week, totalling 50–100 minutes per week, for it to be beneficial to students.

The activities, selected by “Write in Cursive” [46] were built on different levels in order to promote the learning of cursive handwriting:

- Pre-graphism: We started with simple executions that required a progressive degree of motor precision, sign control, directionality, and respect for spacing. In this training, we exerted the subsequent production of cursive letters through repetitive and curvilinear movements.
- Letters Presentation: Letters were presented according to their similarity and gradual articulator movements rather than in alphabetical order, respecting the following sequence: a, o, c, d, g, q / i, t, u / n, m / e, l, f, b / v, w, r, s/ p / h / z / x, y, k, j.
- Connection Between Letters Presentation: Vowels and consonants were shown together. This movement learning becomes more valuable when children start to connect one letter to the next to write digrams and trigrams.

We preferred to use lined exercise books and not quadrille pads to support a progressive motor and space control.

Within each training session, the activities were structured by steps:

- First step: all rules and characteristics of the cursive letters were explained to the entire group by having the teacher demonstrate the correct movements to execute each letter on the blackboard. The teacher dedicated to this activity about 15 minutes of each session.
- Second step: the children subsequently began their individual activity on the pages in the manual. The activity was to copy the cursive letter on a sketch design of the letter either in isolation or at the beginning, middle or end of a word (only the letter presented by the teacher, and not the other letters of the word); this task demanded increasing motor, direction and space control.
- Third step: the next task consisted of copying the same cursive letter on one page of their notebook for about 50 times (A4 format). This task, through repetitive and circular movements, enabled the children to strengthen the writing of cursive letters.
- Fourth step: the last task was the fusion of cursive letters to form syllables and then words. Consequently, children practiced cursive letters writing by controlling the movements and trajectories in order to better understand the relationship between cursive letters, spaces and lines.
- Fifth step: finally, to verify correct production across all students, the teacher performed a double check; the first on-line occurred during the writing of the letters, and consisted of correcting the pupils who produced erratic movements; the second occurred after execution, by verifying the single materials produced by the pupils. Those pupils who showed more uncertain graphic traits or incorrect production were joined by the teacher in the next session.

The training activities did not provide any intervention on reading skills or orthographic knowledge. For these skills, students followed traditional teaching methods.

## Results

### Preliminary analyses

First, we tested for possible associations between the socio-demographic variables (child’s gender and mother’s and father’s years of education) and the variables of interest in the study at each time point. All preliminary analyses were tested using the Bonferroni-corrected alpha level to protect against capitalizing of chance, according to the number of associations that were tested at each time point (6 at the pre-test, and 14 at the post-test and follow-up). None of the measures at each time point was found to be associated with mothers’ and fathers’ years of education. As to the effect of the child’s gender, none of the measures collected at each time point differed between girls and boys, with the exception of the two-letter search in the post-test, t(139) = 3.42, p < .001, with girls performing significantly better compared to boys, M _girls_ = 3.94, SD = 3.10; M _boys_ = 6.36, SD = 4.99 (lower scores indicate a better performance, as scores refer to the number of errors).

### Main analyses

Because we were dealing with a repeated-measures design with measurements collected at two and three points in time (Level 1) nested within cases (Level 2), the aims were tested using multilevel models which allow the treatment of non-independent measures and give the added advantage of being able to deal with missing data at each time point. A set of multilevel models were run, with measures at each time (Level 1) nested within cases (Level 2). Each of the six measures of prerequisite reading and writing skills as well as those of reading, writing and spelling skills (respectively three, two and three measures) was used as the dependent variable. As random effects, we entered intercepts for subjects as well as by-subject random slopes for the effect of time, with a variance components covariance structure. This latter random effect was dropped when it did not result in a significant increase of the model fit. In accordance with the aims, the fixed effects of time and group (intervention vs. control) were tested: the first predictor allowed us to test whether the outcomes underwent a change over time, irrespective of group; the second predictor allowed us to test whether the two groups differed in the outcome measures. Thirdly, the interaction term time X group was inserted in order to verify whether the effect of time was moderated by that of the intervention. The test of our aims depends mainly on this term, which whether significant or not proved that the intervention was causing different growth curves of the outcome/s across the two groups (intervention vs. control). Along with these predictors, in order to control for possible effects on the outcome, fixed effects of the child’s gender were also included in the models and were dropped from the final models if they resulted in non-significant effects. Δ -2LL < .05 and lowest Akaike’s AIC were the fit indexes used to select the models best fitting the data, for nested and non-nested models respectively.

As to the test of the first aim, the models for each outcome measure with the best fit are reported in Table 3: None of the models predicting the prerequisites gained significant fit from the random effects of time, suggesting no significant inter-individual variability in the growth curve of each outcome; therefore, this effect was dropped from each final model. As to the fixed effects of time, results show a linear improvement in the recognition of letters, the search of sequences of letters, and handwriting speed. Conversely, the two-letter search results worsened from one time to the next. Gender was found to be a significant predictor of performance in the two-letter search and the recognition of sequences of letters, with girls performing better compared to boys in both cases. As expected, group condition was found not to be a significant predictor, which means that there were no significant differences between the two groups in the dependent variable, while a significant interaction group X time was found for three out of five outcomes, namely, the performance in the semicircles task, the two-letter search, and handwriting speed. Overall, these interactions mean that over time in the two groups the dependent variables underwent different growth rates. In order to explore these interaction effects and understand the differing growth rates of the measures among the two groups, the mixed models were re-run separately for each sub-group [47]. Each model included the fixed and random effects of time, while the between-subject variance was estimated by entering intercepts for subjects as the random effects. Results showed that performance on the three tasks was better overall among the children belonging to the intervention group compared to those of the control group; as to the performance in the semicircles task, from one time to the next, the children demonstrated reduced errors of b = -1.436, p < .001, intercept = 3.711, p < .001 in the intervention group, compared to b = -.35, n.s., intercept = 3.064, p < .001 in the control group. As to the two-letter-search task, the performance of the control group decreased significantly over time, b = 2.127, p < .001, intercept = 7.247, p < .001, while that of the intervention group remained stable, b = .539, n.s., intercept = 7.386, p < .001. Lastly, as to handwriting speed, the intervention group gained on average almost 16 graphemes per minute across time, compared to the control group which gained on average 11 graphemes per minute from one time point to the next, b = 15.953, p < .001, intercept = 44.641 and b = 11.003, p < .001, intercept = 42.853, p < .001, respectively for each group. Fit of the models run within each group to explore the interaction effects time X group did not improve their fix when estimating the random effects of time, suggesting, therefore, a similar slope for the effects of time among the children of each group (intervention vs. control).

**Table 3:** Mixed models predicting writing and reading prerequisites from time, group (control vs intervention) and gender.

	Semicircles (number of errors)			Recognition of Letters (number of errors)			Search of two letters (number of errors)			Search of sequences of letters (number of errors)			Handwriting Speed (graphemes/minute)		
**Fixed effects**	*b*	*SE*	*df*	*b*	*SE*	*df*	*b*	*SE*	*df*	*b*	*SE*	*df*	*b*	*SE*	*df*
Intercept	3.06***	.258	394.194	16.798***	.708	397.100	42.855***	1.032	372.653	1.032***	.153	393.653	7.025***	.724	393.452
Time (fixed effect)	-.352	.194	279.786	-5.394***	.541	378.752	10.997***	.740	279.643	-.344**	.115	278.857	2.174***	.549	278.570
Group	.647	.359	394.219	-1.690	.985	396.829	1.782	1.440	372.463	-.181	.213	393.678	.476	1.007	393.181
Gender	—	—	—	-1.550*	.645	137.819	—	—	—	—	—	—	-2.323***	.666	138.493
Time × group	-1.084***	.271	280.900	-.006	.753	279.266	4.962***	1.036	280.651	-.071	.161	279.973	-1.650*	.765	279.662
**Random effects**															
σu	.328			.118			1.720			1.168			11.541*		
σe	5.11***			1.801***			40.39***			39.267***			74.399***		
Deviance															
-2LL (df)	1904.399(6)			1470.597(6)			2734.566(7)			2724.750(7)			3046.679(6)		
AIC	1908.399			1474.597			2738.566			2728.750			3050.679		

*p < .05.

**p < .01.

***p < .001.

*Note*. Time: 0,1, 2. Group: 0 = control; 1 = intervention. Gender: male -.5 and female .5. Random effects of time were dropped due to the lack of a significant increase in the fit indexes.

With respect to the test of the second aim, models with the best fit indexes predicting reading skills are reported in Table 4 and show that reading comprehension, fluency and accuracy increase linearly over time and none were predicted by child’s gender; models predicting reading fluency and accuracy also included random effects of time, suggesting significant inter-individual differences in slopes for the effects of time. As to the group effects, the group benefitting from the intervention months before performed better on reading comprehension but worse on reading accuracy when compared to the control group. Lastly, the interaction term group X time was significant for reading comprehension and fluency, which means that over time in the two groups the dependent variables underwent different growth rates. In order to explore these interaction effects and understand the different growth rates of the measures among the two groups, the mixed models were re-run separately for each sub-group [48]. Each model included the fixed and random effects of time, while the between-subject variance was estimated by entering intercepts for subjects as the random effects. As to reading fluency, the model tested among each group showed that the random effects of time increased the fit only for the intervention group, suggesting significant inter-individual variability in the growth curve among the children who had benefitted from the intervention. Over time, the reading fluency of these children decreased significantly, b = -.238, p < .001, intercept = 1.161, p < .001. Conversely, the reading fluency scored of the children in the control group increased significantly over time, b = .198, p < .05, despite having an average starting point lower than that of the intervention group (intercept = 1.131, p < .001).

**Table 4:** Mixed models predicting reading skills from time and group (control vs intervention) and gender.

	Reading comprehension			Reading fluency			Reading accuracy		
**Fixed effects**	*b*	*SE*	*df*	*b*	*SE*	*df*	*b*	*SE*	*df*
Intercept	6.782***	.225	269.363	1.131***	.060	142.000	5.304***	.415	142.000
Time (fixed effect)	1.431***	.297	137.715	.198*	.081	139.160	2.272***	.690	137.817
Group	1.957***	.314	269.363	.029	.084	142.000	-3.201***	.579	142.000
Gender	—	—	—	—	—	—	-2.323***	.666	138.493
Time × group	-1.647***	.415	138.130	-.433***	.113	139.854	-.994	.968	138.252
**Random effects**									
σu	.512			.073**			4.115*		
σe	3.001***			.180**			7.783***		
Var(time)	—			.084			16.503***		
**Deviance**									
-2LL (df)	1140.096(6)			438.420(7)			1588.707(7)		
AIC	1144.096			452.420			1602.707		

*p < .05.

**p < .01.

***p < .001.

*Note*. Time: 0,1, 2. Group: 0 = control; 1 = intervention. Gender: male = -.5; female = .5.

A similar pattern of results emerged from the single slope analysis predicting reading comprehension: the model tested in each group showed that the random effects of time increased the fit only among the intervention group, suggesting a significant inter-individual variability in the slope for the effect of time for the children who had benefitted from the intervention. Over time, these children remained stable in their reading comprehension, b = -213, n.s., intercept = 8.739, p < .001. Conversely, the children of the control group displayed an average level of comprehension lower than that of the intervention group, intercept = 6.782, p < .001, but differently from the intervention children, it increased significantly over time, b = 1.433, p < .001.

Models with the best fit indexes predicting writing skills are reported in Table 5 and show that none of the two indexes for writing fluency was predicted by the child’s gender, and that both increased linearly over time. Besides the fixed effects of time, writing fluency was also predicted by a random effect of time, suggesting significant inter-individual differences in the children’s improvement. Children who had benefited from the intervention had better performance, compared to the control group; nevertheless, as the significant interaction term time X group and the following single slope analysis both suggest, the intervention group started with a higher performance in writing fluency on the word *ONE* which did not increase significantly over time, while the control group displayed a significant increase in the same performance, although having a much lower starting point compared to the former group, b = 1.493, n.s., intercept = 59.972, p < .001 in the intervention group and b = 7.460, p < .001, intercept = 48.797, p < .001.

**Table 5:** Mixed models predicting writing skills from time and group (control vs intervention) and gender.

	Writing Fluency “ONE”			Writing Fluency “NUMBERS’S NAME”		
**Fixed effects**	*b*	*SE*	*df*	*b*	*SE*	*df*
Intercept	48.797***	1.154	265.703	54.318***	1.245	142.000
Time (fixed effect)	7.493***	1.456	139.498	4.523**	1.434	138.017
Group	11.175***	1.610	265.703	3.681*	1.736	142.000
Gender	—	—	—	—	—	—
Time × group	-5.967**	2.039	140.374	3.723	2.011	138.423
**Random effects**						
σu	71.852***			60.025***		
σe	20.104*			46.939***		
Var(time)	—			44.555*		
**Deviance**						
-2LL (df)	2039.196(6)			2101.049(7)		
AIC	2051.196			2115.049		

*p < .05.

**p < .01.

***p < .001.

*Note*. Time: 0,1, 2. Group: 0 = control; 1 = intervention. Gender: male = -.5; female = .5.

Lastly, models with the best fit indexes predicting spelling skills are reported in Table 6 and show that spelling skills were not predicted by gender, nor by random effects of time, suggesting similar slopes among the children. Spelling words and pseudo-words significantly increased over time, while spelling text did not. Children who had benefited from the intervention, when compared to the control children, displayed overall higher performance on all three spelling skills tests, as their performances were characterized by a significantly lower number of mistakes. Lastly, no significant interaction was found between time and group condition, suggesting that both groups underwent the same changes over time.

**Table 6:** Mixed models predicting reading skills from time and group (control vs intervention) and gender.

	Spelling Accuracy for Words (number of errors)			Spelling Accuracy for Pseudowords (number of errors)			Spelling Accuracy for Text (number of errors)		
**Fixed effects**	*b*	*SE*	*df*	*b*	*SE*	*df*	*b*	*SE*	*df*
Intercept	9.710***	.566	254.814	4.478***	.292	258.255	7.275***	.471	260.662
Time (fixed effect)	-2.210***	.675	137.309	-1.401***	.354	138.394	1.103	.580	137.994
Group	-3.751***	.790	254.814	-2.396***	.408	258.255	-3.330***	.657	260.662
Gender	—	—	—	—	—	—	—	—	—
Time × group	-.654	.946	138.140	.494	.496	139.239	-.927	.816	139.318
**Random effects**									
σu	6.703***			1.650**			4.115*		
σe	15.455***			4.260***			7.783***		
Var(time)	—			—			16.503***		
**Deviance**									
-2LL (df)	1637.187(6)			1271.845(6)			1533.535(6)		
AIC	1649.187			1283.845			1545.535		

*p < .05.

**p < .01.

***p < .001.

*Note*. Time: 0,1, 2. Group: 0 = control; 1 = intervention. Gender: male = -.5; female = .5.

## Discussion

This study focused on the impact of visual-motor handwriting training on the reading and writing skills of 6-year-old children. The children involved had not yet begun systematic school handwriting instruction. We therefore aimed to explore the effects of a teaching program focused on intensive cursive instruction on: (a) the prerequisites of writing and reading; and (b) the acquisition of writing and reading skills. The results revealed that changes in reading and writing skills varied as a function of the type of training received. Moreover, within the longitudinal research design we also tested the effects of time. Regarding the first aim, we found that post-training, the intervention group made fewer mistakes than the control group in the “semicircles” task. The intervention group also showed a more stable performance through time in the “two-letter-search task. Moreover, the intervention group was able to write 16 graphemes per minute, while the control group had a rate of 11 graphemes per minute. In line with previous studies, the reading and writing prerequisites strongly correlated with age, thus suggesting that the progressive acquisition of the visual search ability and the semicircles task followed a specific developmental trajectory [45].

We can detect a worsening performance in each group over time in the “two-letter-search” task. Nevertheless, the training group’s performance was more stable than the control group’s performance. This performance difference can be explained by the children’s ability to process the words in their entirety by accessing mental vocabulary, rather than identifying every single letter which forms the word itself (global versus local processing) [45]. As far as writing fluency is concerned, our data comply with previous studies which show a linear relationship between graphic-motor abilities and developmental trajectories [49,50]. The higher number of graphemes written by the intervention group is due to the training. This result is important because handwriting speed can be considered a good predictor for more complex tasks such as orthography and test processing. A substantial gender difference in prerequisite tasks—mainly in analysis and visual search abilities—in favor of females was found. Previous studies have demonstrated a higher rate of learning disabilities in boys than girls, but it has not yet been fully explained why this gender difference appears. In most studies the gender effect appears in the early stages of learning [49]. Therefore, this study suggests that the gender difference can play a relevant role in reading and writing prerequisite skills, but that these differences were no longer present further on when considering writing and reading skills. The second aim of the present study was to analyze the effect of training on reading and writing skills. There are a lot of research studies that demonstrate a linear trend of reading skills, highlighting an increased performance in instrumental reading abilities such as fluency and accuracy, as well as text comprehension. These data can be observed for both groups, but there is also an inter-individual difference over time. This variability affects the first learning phases in reading; performances become more homogeneous with schooling and with age. Accuracy reading performance in the control group increased more than in the intervention group, since there were substantial differences at the beginning: the control group started from a significantly lower average performance, not due to an effect of the training. Similarly, at the early learning stages, text comprehension processes are necessarily distinguished by a huge inter-individual variability, because text comprehension is a complex learning process in which several abilities merge; as a consequence, it takes a longer period and more skills to make this ability stable over time. In the early learning stages, we cannot find a strict correlation between reading abilities and comprehension [50]; indeed, some studies show that children in the fourth grade are able to understand the meaning of a text even without proper accuracy abilities [51]. This result may be obtained by submitting simpler texts with lower syntactic complexity [5]. It is worth pointing out how the training produced a certain stabilizing effect from the early learning stages, for both instrumental reading abilities and text comprehension. In various studies, this fact is seen as a good predictor of study skills in the following years. Concerning writing and reading abilities, there is a remarkable linear growth over the time due to a higher grapho-motor control. Concerning writing speed, many studies show that automating certain activities in the act of handwriting may enable students to apply their cognitive resources to more complex activities, such as orthographic accuracy [27]. As proof of what was previously stated, the intervention group achieved better performance both in orthographic ability and text fundamental units. These findings are in agreement with the literature in affirming that the development of more fluent writing with grapho-motor abilities during the early stages of learning to write enables students to reach better accuracy levels for orthographic features [48]. The most interesting result related to cursive handwriting training is the data regarding writing fluency. A great deal of the literature supports the idea that children with more fluent handwriting in the early stages of learning show better writing abilities in terms of orthography and increased text composition skills. Our results support the literature by underlining the relationship between graphic and orthographic skills. This relationship is observed and supported by other studies [15,16,20,25], which show the contribution made by this variable with regard to more complex cognitive writing skills. We also observed that the intervention group’s handwriting skills changed dramatically over the school year, showing better results than those predicted by the usual evolutionary trends. These results demonstrate how children can improve not only basic skills, but also subsequent learning abilities thanks to domain-specific training carried out in the field of grapho-motor learning. Our study supports recent works that demonstrate how improvements in instrumental handwriting features may occur upon teaching and direct, explicit daily practice [15,16], particularly during the early stages of schooling. All this suggests the importance of automating the production of letters and words during writing so children can direct their attentive resources to the regulation of more complex aspects of text production, such as the decoding of texts and the orthographic accuracy of writing [8]. In fact, working memory seems to play a key role in the processes of writing and reading.

Bourdin and Fayol [7] examined writing processes within the explicit context of working memory. They varied the response modality (spoken vs. written) in a serial recall task and found that recall was significantly poorer in the written condition for children but not for adults. The authors interpreted these findings as evidence that the transcription process of adults, but not children, was sufficiently fluent to operate with minimal working memory demands. When adults were required to write in cursive uppercase letters, thereby preventing their use of overlearned, highly fluent transcription processes and depriving them of access to working memory, also adults showed poorer recall when writing. In a related series of experiments, Bourdin and Fayol [7] changed the task from serial recall to sentence generation and again demonstrated that transcription imposed resource costs for children but not for adults. Thus, until transcription processes develop sufficient fluency, writers seem constrained by working memory limits [9]. With regard to the reading processes, certainly the training of visuo-spatial skills has strengthened the positive effect that writing has provided to reading skills. A study undertaken at Indiana State University, in which an experimental group of children were taught exclusively cursive writing in the first grade, appears to support our position. Achievement in spelling and word reading was higher in the experimental group, while there were more reversals and transposition errors in the control group [52]. Recent studies support the same results [30,53,54].

Nevertheless, we believe that working on the quality of the practice is fundamental; otherwise it would be highly improbable for writing feature rates to increase without negatively affecting readability. Concerning this feature of education, further investigation is needed to better understand the relation between handwriting practice and the development of writing abilities during primary school. Moreover, our study introduces an innovative fact not previously dealt with in recent literature: that children who adopted the cursive type as the only handwriting type showed a higher writing rate than pupils using more types. This fact contrasts with the literature which states that the cursive type decreases writing rates [51]. We also observed that pupils using cursive as the only handwriting type had better results in producing orthographically correct words than students using more types. As shown by other studies [20], it seems that the grapho-motor component affects word production management, especially for writers in the learning phase. In addition, we observed that children who only learned the cursive type made faster improvements in reading. This fact may be explained by a major focus of active resources on the lexical access task. The very nature of the cursive type may help students to easily memorize and recall a word unit, since in the cursive type the letters of a word are linked one to another, while in print type they are separated [35].

In conclusion, like other studies [10,11,35], our work tends to demonstrate how, upon training, writing and reading abilities improve in terms of written letter rate (students write faster), orthography (words are written correctly), and reading (students read and understand better). However, writing quality is a parameter to be investigated thoroughly in further studies. Considering writing type, we can observe how students who learn every type simultaneously do not achieve results as good as those achieved by cursive-only students. This finding supports the idea that the development of writing abilities in primary school is better favored by the teaching of a single type of handwriting, namely cursive handwriting. Furthermore, teaching of the cursive type generates improvement in graphic and orthographic word production by the end of the school year. A remarkable feature to be taken into account is the rapid improvement of basic skills in the intervention group as compared to the control group.

Our research sheds light on a number of educational issues. Firstly, it is necessary to think about the role of grapho-motor abilities in the development of handwriting skills, as well as giving more weight to grapho-motor skills in teaching plans. Secondly, it is important to support the teaching community to ensure that decisions regarding handwriting automatization are taken at the beginning of the educational process [55]. In order to do so, explicit and direct teaching of letter shapes and frequent practice are essential elements [35]. Last but not least, it is necessary to think further about the relevance of single-learning process based teaching, since it has been demonstrated that by acting on single learning abilities, there are greater advantages to be had in future learning.
